# Bacterial and Fungal Communities Associated with the Ectomycorrhizospheric Soil and Stem Endosphere of the Mycoheterotrophic Plant *Monotropa uniflora*

**DOI:** 10.3390/plants15081145

**Published:** 2026-04-08

**Authors:** Leandro Alberto Núñez-Muñoz, Brenda Yazmín Vargas-Hernández, Melissa Cheryn García-Sierra, Berenice Calderón-Pérez, Beatriz Xoconostle-Cázares, Roberto Ruiz-Medrano

**Affiliations:** 1 Departamento de Biotecnología y Bioingeniería, Centro de Investigación y de Estudios Avanzados, Av. Instituto Politécnico Nacional 2508, Col. San Pedro Zacatenco, Mexico City 07360, Mexico; leandro.nunez@cinvestav.mx (L.A.N.-M.); byvargas@cinvestav.mx (B.Y.V.-H.); melissa.garcia@cinvestav.mx (M.C.G.-S.); bcalderon@cinvestav.mx (B.C.-P.); 2 Programa de Doctorado Transdiciplinario en Desarrollo Científico y Tecnológico para la Sociedad, Centro de Investigación y de Estudios Avanzados, Av. Instituto Politécnico Nacional 2508, Col. San Pedro Zacatenco, Mexico City 07360, Mexico

**Keywords:** microbial community structure, metabarcoding, plant-associated microbiome, mycorrhizosphere, compartmentalization, fungal-bacterial interactions

## Abstract

The mycoheterotrophic plant *Monotropa uniflora* relies on fungal symbionts for carbon and nutrient acquisition. However, its interactions with other microbial groups, beyond ectomycorrhizal fungi, remain unexplored. Here, we characterized bacterial and fungal communities associated with *M. uniflora* across two compartments: ectomycorrhizospheric soil linked to the mycorrhizal network and the surface-sterilized lower stem endosphere. Microbial community composition was assessed using high-throughput amplicon sequencing of the bacterial 16S rRNA gene and the fungal ITS region. Fungal richness was consistently higher in ectomycorrhizospheric soil than in the stem endosphere, whereas bacterial alpha diversity showed no consistent differences between compartments. Multivariate analyses suggested compartment-associated patterns in both bacterial and fungal community composition. Ectomycorrhizospheric soil was dominated by saprotrophic fungal taxa and bacterial groups with predicted metabolic potential, including taxa associated with iron, sulfur and nitrogen cycling. In contrast, the lower stem endosphere was enriched in bacterial taxa commonly associated with anaerobic and nitrogen-related metabolisms. Functional predictions further suggested an increase of carbon fixation-related pathways in rhizosphere-associated bacterial communities. Together, these results indicate that *M. uniflora* is associated with distinct and structured microbial assemblages across soil and internal plant compartments, highlighting the predicted functional potential of bacterial communities in nutrient- and carbon-related processes in mycoheterotrophic plant–soil systems alongside fungal partners.

## 1. Introduction

Mycoheterotrophy is a specialized evolutionary strategy in obtaining carbon from associated fungi rather than through photosynthesis. This mode of nutrition has evolved repeatedly across land plants and is found in diverse lineages, including liverworts, lycophytes, ferns, angiosperms, and gymnosperms. More than 23,000 plant species exhibit mycoheterotrophy during some stage of their life cycle, and at least 514 angiosperms are fully mycoheterotrophic, relying entirely on fungal carbon throughout development [1,2]. *Monotropa uniflora* (Ericaceae), commonly known as ghost plant or Indian pipe, is a fully mycoheterotrophic species that acquires carbon and nutrients exclusively through monotropoid mycorrhizal associations with Russulaceae fungi, primarily *Russula* and *Lactarius* spp. [3,4].

Like other plants, *M. uniflora* exists within a complex microbial environment in which fungi, bacteria, and the host constitute an integrated ecological unit or holobiont [5]. In autotrophic plants, the microbiome contributes to nutrient mobilization, pathogen suppression, hormone regulation, and tolerance to abiotic stress [6,7]. However, the extent to which these microbial functions operate or are reconfigured in full mycoheterotrophic species remains largely unknown. Because *M. uniflora* does not photosynthesize, exhibits reduced root structures, and depends entirely on mycorrhizal networks for carbon acquisition, the composition and functional roles of its associated microbiota are likely to diverge from those of autotrophic hosts [8].

*M. uniflora* is typically found in cool to temperate, shaded forests across the Northern Hemisphere, commonly occurring in coniferous and mixed woodlands dominated by *Fagus*, *Pinus*, *Quercus* and *Salix* trees [9]. Its distribution is tightly linked to soil properties, forest structure, and the availability of compatible Russulaceae species [10]. As a secondary heterotroph drawing carbon indirectly from surrounding trees via fungal intermediaries, *M. uniflora* participates in tripartite interactions (tree, mycorrhizal fungus, and plant) that shape belowground carbon pathways and may influence local microbial community structure [11]. Despite this ecological importance, research has focused on identifying fungal symbionts from root tips [12], while much less attention has been given to the broader microbial communities inhabiting its rhizosphere or internal tissues.

The rhizosphere of autotrophic plants is structured largely by root exudates that supply carbon and shape microbial recruitment, with up to 20% of photosynthetic products released into the soil [13]. In contrast, mycoheterotrophic plants obtain carbon through fungal intermediaries rather than photosynthesis, a difference likely to influence rhizospheric and endophytic microbial communities and host-microbe interactions. In this context, endophytes associated with *M. uniflora* may contribute to processes related to nutrient acquisition.

To investigate the microbial communities associated with *M. uniflora*, amplicon-based sequencing techniques, targeting the 16S ribosomal RNA (rRNA) gene and the internal transcribed spacer (ITS) region for bacterial and fungal identification were employed. The 16S rRNA gene is a highly conserved marker that provides effective phylogenetic resolution of bacterial taxa, whereas the ITS region is recognized as the standard barcode for most fungal species due to its interspecific variability [14]. These genetic markers facilitate high-throughput, culture-independent profiling of microbial communities and are particularly advantageous for examining complex and poorly characterized microbiomes.

Although *M. uniflora* possesses significant ecological and evolutionary importance, the diversity and functional potential of its associated bacterial and fungal communities remain poorly understood, especially beyond the mycorrhizal associations. In particular, it remains unclear whether distinct plant-associated compartments harbor different microbial assemblages and how bacterial and fungal communities are structured within these habitats. Given the contrasting carbon sources and microhabitats of mycoheterotrophic systems, we hypothesized that microbial communities in *M. uniflora* are structured in a compartment-specific manner. Specifically, we predicted that ectomycorrhizospheric soil (ECT) and the lower stem endosphere (END) host distinct assemblages. To test this hypothesis, we characterized bacterial and fungal communities in ECT and END of *M. uniflora* using amplicon sequencing of bacterial 16S rRNA and fungal ITS markers. By comparing these two compartments, our study aims to identify microbial taxa associated with this highly specialized nutritional strategy and to provide ecological context for host–microbe interactions in a fully mycoheterotrophic plant. This work expands our understanding of belowground microbial ecology in forest ecosystems and highlights microbial contributors to plant nutrition and survival in non-photosynthetic plant systems.

## 2. Results

**Figure 1:**
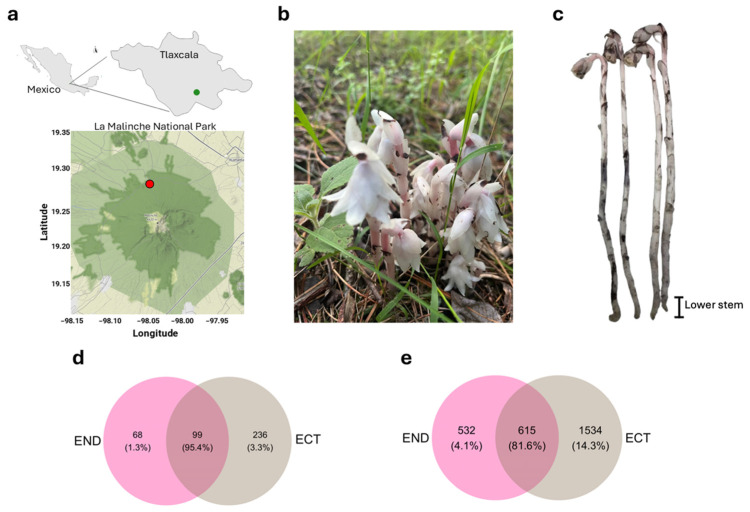
Sampling site, organism under study, and shared ASV analysis. (**a**) Location of the sampling site in La Malinche National Park, Tlaxcala, Mexico (red dot indicates the sampling location). (**b**) Individuals of *M. uniflora* at the sampling site. (**c**) Sampling of the lower stem segments used for genomic DNA extraction. Venn diagrams show shared and unique ASVs between lower stem endosphere (END) and ectomycorrhizospheric soil (ECT) communities for fungal (**d**) and bacterial (**e**) communities. Scale bar = 2 cm.

Samples of *M. uniflora* were collected in La Malinche National Park, Mexico (Figure 1a,b). Two compartments were defined for microbiome analyses: the ECT and END. For END samples, lower stem tissue (Figure 1c) was excised and surface-sterilized prior to DNA extraction. High-throughput amplicon sequencing of bacterial (16S rRNA) and fungal (ITS) markers from ECT and plant END associated with *M. uniflora* generated a total of 527,278 paired-end 16S rRNA reads and 604,309 ITS reads (Table S1). After quality filtering, denoising, and chimera removal, 345,924 high-quality bacterial reads and 401,770 fungal reads were retained. Bacterial reads ranged from 29,415 to 72,168 per sample (mean = 57,654), yielding 2717 amplicon sequence variants (ASVs), while fungal reads ranged from 39,873 to 87,045 per sample (mean = 66,962), resulting in 505 ASVs. Rarefaction curves reached saturation for all samples, indicating sufficient sequencing depth; therefore, all samples were included in downstream analyses (Figure S1). For fungi, 99 ASVs (95.4%) were shared between compartments, whereas 68 (1.3%) and 236 (3.3%) ASVs were unique to END and ECT, respectively (Figure 1d). In contrast, bacterial communities showed larger differentiation as 615 ASVs (81.6%) were shared, while 532 (4.1%) and 1534 (14.3%) ASVs were unique to END and ECT, respectively (Figure 1e).

**Figure 2:**
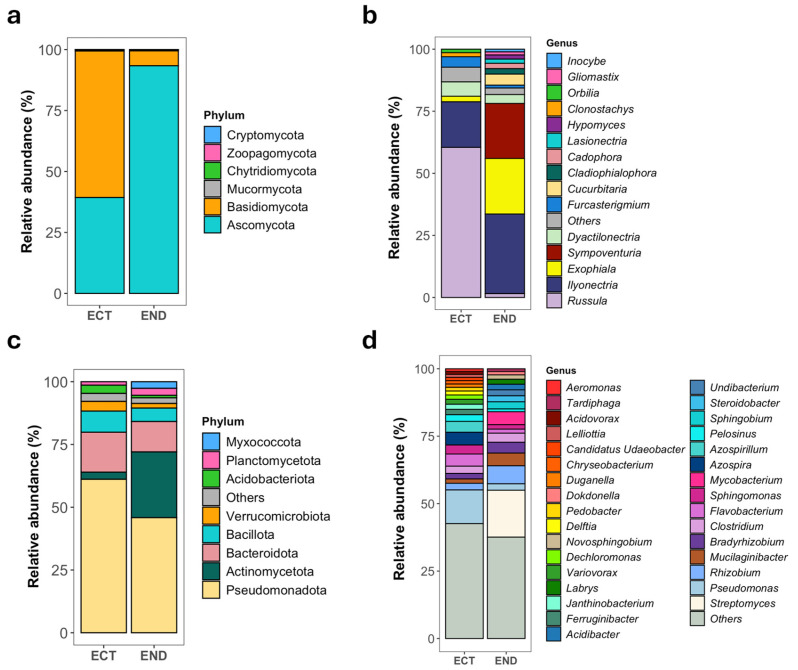
Relative abundance of fungal and bacterial communities in *M. uniflora*. Bar plots show the composition of fungal (**a**,**b**) and bacterial (**c**,**d**) communities at the phylum (**a**,**c**) and genus (**b**,**d**) levels across two compartments: lower stem endosphere (END) and ectomycorrhizospheric soil (ECT). In genus-level bar plots, taxa representing <1% relative abundance were grouped as “Others”.

Fungal communities encompassed six phyla and 106 genera. In the END samples, the community was dominated by Ascomycota (93.89%) and Basidiomycota (5.64%), while Mucormycota, Zoopagomycota, Chytridiomycota, and Cryptomycota were detected at lower relative abundances (<0.5% combined). In contrast, ECT samples were dominated by Basidiomycota (55.72%) and Ascomycota (43.72%), with other phyla again present at low abundance (Figure 2a). At the genus level, END fungal communities were represented by *Ilyonectria*, *Sympoventuria*, and *Exophiala*, together accounting for more than 75% of the relative abundance, followed by several additional ascomycete genera each contributing to less than 5%. In ECT samples, *Russula* was the dominant genus (55.91%), followed by *Ilyonectria* and a set of lower-abundance taxa including *Dactylonectria*, *Furcasterigmium* and *Exophiala*, with numerous additional genera contributing <2% relative abundance (Figure 2b).

Bacterial communities comprised 32 phyla and 349 genera. END samples were dominated by Pseudomonadota (42.76%) and Actinomycetota (29.47%), followed by Bacteroidota (10.41%) and Bacillota (7.29%). Additional phyla including Planctomycetota, Myxococcota, Verrucomicrobiota, and Acidobacteriota were detected at lower relative abundances. In ECT samples, Pseudomonadota remained dominant (61.94%), but Bacteroidota (15.13%) and Bacillota (9.26%) increased in relative abundance, while Actinomycetota declined (2.61%). Soil-associated phyla such as Acidobacteriota and Verrucomicrobiota were more prominent in ECT than in END (Figure 2c). At the genus level, END communities were characterized by a mixture of putative endophytes and metabolically versatile taxa, with *Streptomyces*, *Mycobacterium*, *Rhizobium*, *Bradyrhizobium*, *Clostridium*, and *Mucilaginibacter* among the most abundant genera. Additional genera associated with nitrogen metabolism, anaerobic processes, or secondary metabolite production were consistently detected at lower abundances. In contrast, ECT communities were dominated by genera commonly associated with rhizosphere and soil environments, including *Pseudomonas*, *Azospira*, *Azospirillum*, *Flavobacterium*, *Sphingomonas*, *Pelosinus*, *Clostridium*, and *Rhizobium*, alongside numerous lower-abundance taxa linked to nitrogen, iron, and sulfur cycling (Figure 2d). The complete taxonomic profiles of fungal and bacterial communities are provided in File S1.

Analysis of taxonomic exclusivity revealed distinct compartment-specific patterns. Two bacterial phyla, five classes, ten orders, 19 families and 38 genera (including *Frankia*, *Aquisphaera*, *Agrobacterium*, *Azorhizobium* and *Paludisphaera*) were uniquely detected in END samples (File S1, bold text). On the other hand, the ECT contained a broader set of exclusive bacteria, comprising seven phyla, 13 classes, 25 orders, 48 families and 85 genera. For fungal communities, exclusivity patterns were more limited. No fungal phyla were uniquely associated with the END compartment; however, one class, two orders, five families, and 11 genera (such as *Gymnostellatospora*, *Hyalorbilia*, *Hypholoma*, *Cordyceps* and *Udeniozyma*) were detected exclusively among endophytes. In contrast, the ECT compartment harbored a larger number of exclusive fungal taxa across higher and lower ranks, comprising seven classes, 16 orders, 38 families and 49 genera.

**Figure 3:**
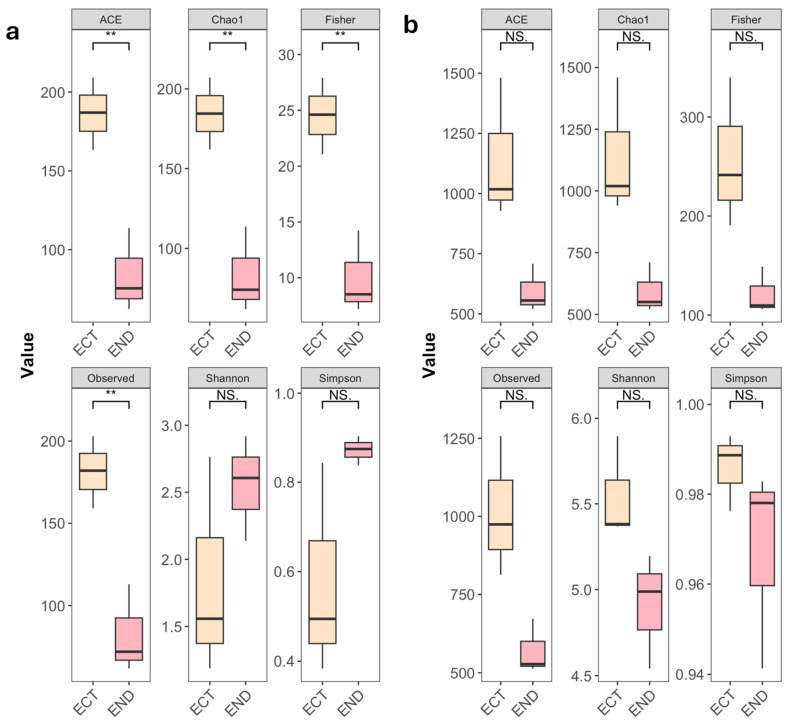
Alpha diversity indices of fungal (**a**) and bacterial (**b**) communities in ectomycorrhizospheric soil (ECT) and lower stem endosphere (END) samples of *M. uniflora*. Boxplots depict alpha diversity metrics (ACE, Chao1, Fisher, Observed, Shannon, and Simpson) for fungal and bacterial communities across ECT and END. Significant differences (*p*  <  0.01) are indicated with asterisks (**), while non-significant comparisons are labeled as “NS”.

Differences in alpha diversity between compartments were evaluated using the Wilcoxon rank-sum test. For fungal communities, richness-based indices showed significantly higher values in the ECT compared to the END compartment, including ACE (*p* = 0.0061), Chao1 (*p* = 0.006), Fisher’s alpha (*p* = 0.0064), and Observed richness (*p* = 0.008) (Figure 3a). In contrast, no significant differences were observed for the Shannon (*p* = 0.28) and Simpson (*p* = 0.17) indices, suggesting similar community evenness. For bacterial communities, ACE (*p* = 0.063), Chao1 (*p* = 0.065), Fisher’s alpha (*p* = 0.056), Observed richness (*p* = 0.066), Shannon diversity (*p* = 0.060) and Simpson diversity (*p* = 0.280) did not differ significantly between compartments (Figure 3b).

**Figure 4:**
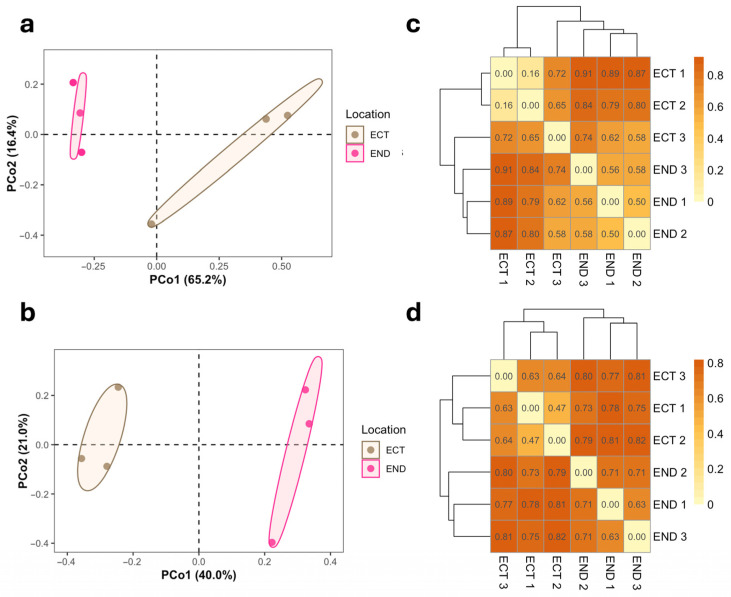
Beta diversity of fungal and bacterial communities associated with the ectomycorrhizospheric soil (ECT) and lower stem endosphere (END) of *M. uniflora*. Principal Coordinates Analysis (PCoA) plots based on Bray–Curtis dissimilarities show compartment-associated patterns in community composition, shown with 95% confidence ellipses. The first two coordinates explain 81.6% of the variance for fungi (**a**) and 61.0% for bacteria (**b**). Corresponding heatmaps show pairwise Bray–Curtis dissimilarities among fungal (**c**) and bacterial (**d**) taxa, accompanied by hierarchical clustering.

Beta diversity was assessed using Bray–Curtis dissimilarities and visualized by Principal Coordinates Analysis (PCoA). For both fungal and bacterial communities, ordination plots suggested a tendency for samples to cluster according to compartment (ECT vs. END) (Figure 4a,b). This pattern was further supported by hierarchical clustering of Bray–Curtis dissimilarities, which grouped samples by compartment (Figure 4c,d). In line with these patterns, ANOSIM showed high R values for both bacterial (R = 0.963) and fungal (R = 0.852) communities; however, given the limited number of biological replicates, these results were interpreted cautiously. In contrast, PERMANOVA did not detect statistically significant differences in community structure between compartments (fungi: R^2^ = 0.58, *p* = 0.10; bacteria: R^2^ = 0.39, *p* = 0.10). To evaluate whether differences in community composition could be influenced by unequal within-group dispersion, we performed a beta-dispersion analysis based on Bray–Curtis distances. No significant differences in dispersion were detected between compartments for either bacterial (PERMDISP, F = 1.05, *p* = 0.40) or fungal communities (PERMDISP, F = 0.31, *p* = 0.70), indicating comparable within-group variability between END and ECT samples (Figure S2).

**Figure 5:**
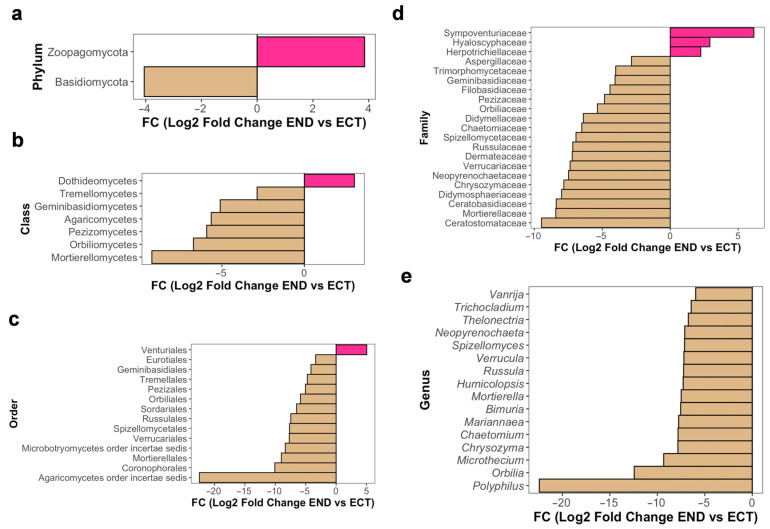
Differential abundance patterns of fungal taxa between ectomycorrhizospheric soil (ECT) and lower stem endosphere (END) compartments of *M. uniflora* across taxonomic ranks. Bar plots show the log2 fold change (END vs. ECT) of fungal taxa identified as significantly different in abundance (adjusted *p* < 0.05) using DESeq2 at the phylum (**a**), class (**b**), order (**c**), family (**d**) and genus (**e**) levels. Negative values (beige) indicate taxa with higher relative abundance in ECT, whereas positive values (pink) indicate taxa with higher relative abundance in END.

Differential abundance analysis was performed to identify the microbial taxa showing differences in relative abundance between the END and ECT compartments. In fungal communities, several taxa exhibited significant differences across multiple taxonomic levels. At the phylum level, Zoopagomycota was more abundant in END samples, whereas Basidiomycota was enriched in ECT (Figure 5a). Sympoventuriaceae was identified as differentially accumulated and this pattern was also reflected at higher taxonomic ranks, including the order Venturiales and the class Dothideomycetes (Figure 5b,c). At the family level, Sympoventuriaceae, Hyaloscyphaceae and Herpotrichiellaceae were differentially abundant in END tissue, although their taxonomic resolution could not be refined to the genus level (Figure 5d). At the genus level, no taxa were significantly enriched in END; however, several genera were more abundant in ECT, including *Polyphilus*, *Orbilia*, *Microthecium*, *Chrysozyma*, *Chaetomium*, *Mariannaea*, *Bimuria*, *Mortierella*, *Humicolopsis*, *Russula*, *Verrucula*, *Spizellomyces*, *Neopyrenochaeta*, *Thelonectria*, *Trichocladium*, and *Vanrija* (Figure 5e). These compartment-associated patterns were further supported by the clustering of variance-stabilized abundances of differentially enriched genera across samples (Figure S3a).

**Figure 6:**
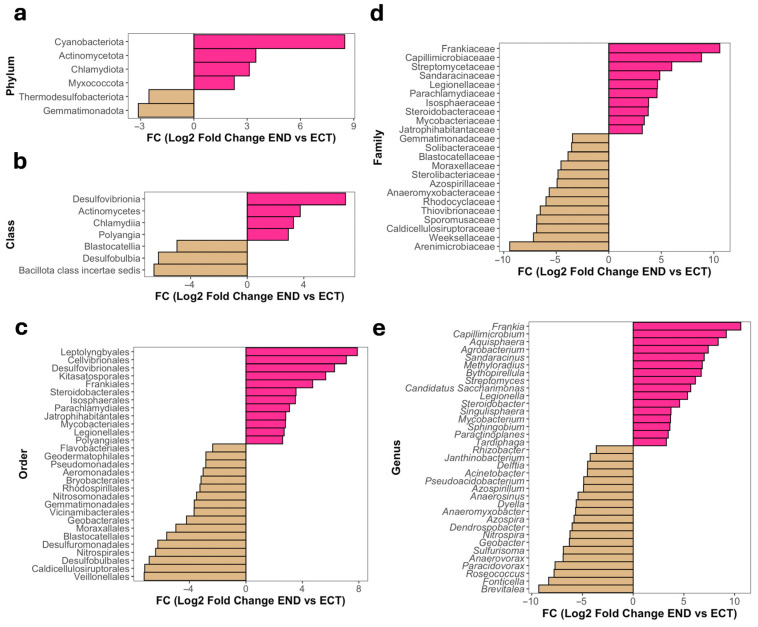
Differential abundance patterns of bacterial taxa between ectomycorrhizospheric soil (ECT) and lower stem endosphere (END) compartments of *M. uniflora* across taxonomic ranks. Bar plots show the log2 fold change (END vs. ECT) of bacterial taxa showing statistically supported differences in relative abundance (adjusted *p* < 0.05) using DESeq2 at the phylum (**a**), class (**b**), order (**c**), family (**d**) and genus (**e**) levels. Negative values (beige) represent taxa with higher relative abundance in ECT, while positive values (pink) indicate taxa with higher relative abundance in END.

In contrast, bacterial communities exhibited a more balanced pattern of differential relative abundance between compartments. At the phylum level, Cyanobacteriota, Actinomycetota, and Chlamydiota showed higher relative abundance in the END compartment, while Gemmatimonadota and Thermodesulfobacteriota were more abundant in ECT (Figure 6a). At the class level, Desulfovibrionia, Actinomycetes, Chlamydia and Polyangia were enriched in END, whereas Bacillota *incertae sedis*, Desulfobulbia and Blastocatellia showed higher relative abundance in ECT (Figure 6b). At the order level, several actinobacteria orders, including Leptotrichiales, Cellvibrionales and Frankiales, exhibited higher relative abundance in END samples, whereas Calditrichales, Veillonellales, and Nitrospirales were enriched in ECT (Figure 6c). At the family level, Frankiaceae, Capillimicrobiaceae, and Streptomycetaceae showed higher relative abundance in END, while Arenimicrobiaceae, Weeksellaceae, and Caldicellulosiruptoraceae were enriched in ECT (Figure 6d). At the genus level, multiple taxa showed higher relative abundance in END, including *Frankia*, *Capillimicrobium*, *Aquisphaera*, *Agrobacterium*, *Sandaracinus*, *Methyloradius*, *Bythopirellula*, *Streptomyces*, *Candidatus Saccharimonas*, *Legionella*, *Steroidobacter*, *Singulisphaera*, *Mycobacterium*, *Sphingobium* and *Paractinoplanes*, while *Brevitalea*, *Fonticella*, *Roseococcus*, *Paracidovorax*, *Anaerovorax*, *Sulfurisoma*, *Geobacter*, *Nitrospira*, *Dendrospobacter*, *Azospira*, *Anaeromyxobacter*, *Dyella*, *Anerosinus*, *Azospirillum*, *Pseudoacidobacterium*, *Acinetobacter*, *Delftia*, *Janthinobacterium* and *Rhizobacter* showed higher relative abundance in ECT samples (Figure 6e). These differences are reflected in the clustering of samples and taxa based on variance-stabilized abundances (Figure S3b).

**Figure 7:**
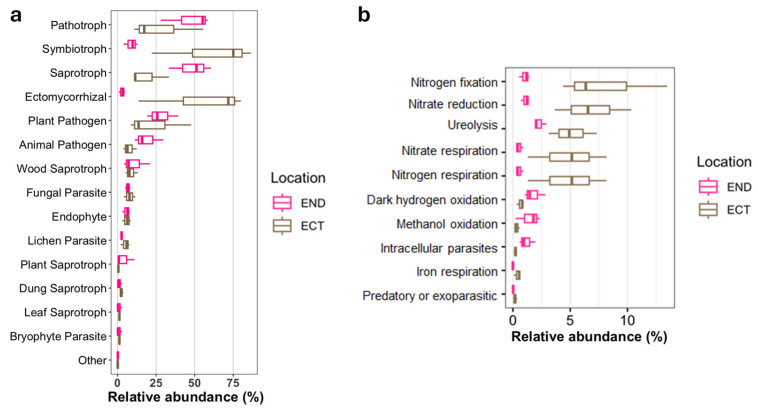
Predicted functional profiles of microbial communities in ectomycorrhizospheric soil (ECT) and lower stem endosphere (END) samples of *Monotropa uniflora*. (**a**) Relative abundances of fungal trophic modes and ecological guilds predicted by FUNGuild based on taxonomic annotation. (**b**) Relative abundances of inferred functional potentials of bacterial communities based on FAPROTAX annotations.

Functional profiling of fungal communities revealed marked differences between ECT and END samples. ECT communities were dominated by saprotrophic fungi, particularly with dung and litter saprotrophs, which may be consistent with a higher representation of decomposer-associated taxa in the surrounding soil. In contrast, END communities displayed higher proportions of ectomycorrhizal fungi and plant pathogens, suggesting a shift toward taxa associated with symbiotic and potentially pathogenic lifestyles within plant tissues (Figure 7a). Additionally, predicted bacterial functional profiles indicated that taxa associated with nitrogen-related processes such as nitrogen fixation, nitrate reduction, and ureolysis were significantly more abundant in ECT, suggesting a greater representation of taxa associated with nitrogen cycling in this compartment (Figure 7b).

**Figure 8:**
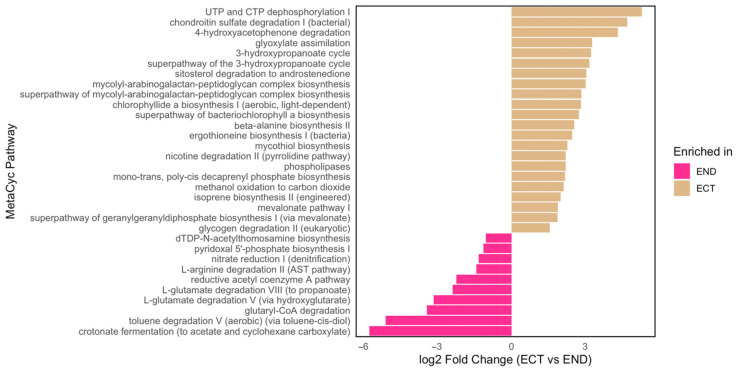
Differentially abundant bacterial functional pathways predicted by PICRUSt2 between ectomycorrhizospheric soil (ECT) and lower stem endosphere (END) samples. Bar plot showing bacterial metabolic pathways with significant differences in relative abundance between ECT and END, based on PICRUSt2-predicted metabolic pathways. Pink bars represent pathways enriched in END, whereas beige bars represent pathways enriched in ECT. Functional annotations correspond to MetaCyc pathways, and the x-axis represents the log2 fold change (ECT vs. END).

Functional prediction using PICRUSt2 v2.4.2 revealed significant differences in predicted bacterial metabolic pathways between ECT and END samples. Pathways related to polyamine biosynthesis, glutamate degradation, and nitrate reduction were significantly enriched in END, consistent with a predicted increase in nitrogen-associated and stress-related metabolic potential within plant tissues. In contrast, glyoxylate assimilation and the 3-hydroxypropanoate cycle were more abundant in ECT, suggesting a higher predicted representation of pathways related to carbon assimilation and alternative energy metabolism in the surrounding soil environment (Figure 8).

To characterize the edaphic conditions and nutritional context associated with the growth of *M. uniflora*, physicochemical and nutritional analysis were performed on rhizospheric soil and plant tissue. The rhizospheric soil exhibited a loam texture, moderately acidic pH (6.62), and low electrical conductivity (0.10 dS/m), indicating non-saline conditions. Organic matter content was high (7.10%), and macronutrient concentrations were elevated, particularly phosphorus (243.96 mg/kg), potassium (2064 mg/kg), calcium (5865 mg/kg), and magnesium (864 mg/kg). Micronutrient levels, including iron (92.63 mg/kg), zinc (8.91 mg/kg), and manganese (56.87 mg/kg) were also quantified. Additionally, the soil displayed low bulk density (0.53 g/cm^3^), suggesting high porosity and favorable conditions for aeration and root penetration (Table S2).

The nutrient analysis of *M. uniflora* tissue revealed high concentrations of potassium (3.85%) and calcium (7.63%), both exceeding typical ranges reported for most plant species. Nitrogen (2.01%) and phosphorus (0.24%) were detected at moderate levels, while magnesium (1.73%) was comparatively high. Among micronutrients, iron showed an exceptionally high concentration (1449.75 mg/kg). Zinc (77.50 mg/kg) and manganese (52.50 mg/kg) were also present at elevated levels, while copper (18.25 mg/kg) and boron (26.24 mg/kg) remained within expected physiological ranges (Table S3).

## 3. Discussion

Differential abundance analyses identified compartment-associated microbial signatures across the compartments of *M. uniflora*. The ECT compartment was characterized by a higher relative abundance of saprotrophic taxa commonly associated with decomposition and nutrient recycling in organic-rich forest soils. *Polyphilus* emerged as the most differentially abundant fungal genus and has previously been reported in association with plant roots, parasitic nematodes, and truffles [15]. Additional enriched taxa included *Orbilia*, known for saprotrophic and nematophagous lifestyles [16], and *Microthecium*, frequently detected in dung, plant debris, and conifers, where it may occur as a fungal parasite or endophyte [17]. These patterns suggest that the ECT may harbor fungal assemblages associated with organic matter turnover rather than direct host colonization.

On the other hand, no fungal genera were significantly enriched in the END compartment. However, this fungal community was dominated by Ascomycota, with genera such as *Ilyonectria*, *Sympoventuria*, and *Exophiala*. These taxa belong to fungal lineages reported as plant-associated fungi, including endophytes or tissue-latent colonizers [18,19,20]. Their prevalence in *M. uniflora* suggests that the endosphere functions as a selective microhabitat, favoring microorganisms adapted to host-derived conditions including restricted oxygen availability, plant defense compounds, and limited nutrient diffusion. In contrast to the ECT, the END compartment exhibited reduced diversity, consistent with host filtering processes commonly observed in plant endospheres [21,22]. In mycoheterotrophic plants, such endophytes may participate in microbial competition, nutrient transformation, or host protection, although their ecological roles remain largely unresolved. The presence of exclusive endophytic taxa in the END compartment may be consistent with processes such as host filtering, early-life acquisition, symbiont-mediated entry and long-term persistence within internal tissues [23].

Bacterial communities exhibited compartment-associated trends, consistent with distinct physicochemical and ecological conditions between END and ECT. The END was enriched in genera such as *Methyloradius*, *Sandaracinus*, *Capillimicrobium*, and *Aquisphaera*, taxa commonly associated with anaerobic and redox-sensitive metabolisms, which may be compatible with the low-oxygen microenvironments expected within non-photosynthetic plant tissues [24,25,26]. Genera such as *Capillimicrobium* and *Streptomyces*, enriched in END, have also been reported in diverse soil and plant-associated microbial communities, where members of these taxa are known to possess several metabolic capabilities, including the biosynthesis of secondary metabolites [27,28,29]. However, direct functional activity cannot be inferred from amplicon sequencing data alone. In contrast, the ECT was enriched in taxa associated with diverse biogeochemical processes, including *Geobacter*, *Sulfurimonas*, *Desulfovibrio*, *Anaeromyxobacter*, and *Anaerovorax*. These genera are reported to participate in iron and sulfur redox cycling [30,31], which may be consistent with the presence of microoxic or anoxic microsites within the ECT that support metabolically diverse respiratory strategies. Additional taxa enriched, including *Dyella*, *Delftia*, *Acinetobacter*, and *Janthinobacterium*, have been reported in rhizospheric or endophytic contexts and are associated with functions such as metal transformation [32,33], antimicrobial compound production [34], and stress tolerance [35], suggesting that ECT may represent a metabolically versatile and competitive environment.

Nitrogen cycling appeared to vary between compartments. In the ECT, *Nitrospira* may indicate taxa commonly associated with nitrification [36], while multiple enriched genera (*Azospira*, *Azospirillum*, *Anaeromyxobacter*, *Geobacter*) include taxa with reported nitrogen-fixation capability [37]. Additional taxa (*Dyella*, *Delftia*, *Acinetobacter*) harbor nitrogenase genes and may potentially contribute to nitrogen transformations [38]. In END, taxa with reported diazotrophic potential, including *Rhizobium*, *Bradyrhizobium*, *Clostridium*, *Pseudomonas*, *Sphingomonas*, *Novosphingobium*, *Flavobacterium*, *Pelosinus*, *Dokdonella* and *Frankia* were detected, suggesting that nitrogen-fixing capacity may be distributed across both compartments [39,40]. However, these inferences are based on taxonomic associations and predictive functional tools and should be interpreted as potential rather than demonstrated activity. Similar patterns have been reported in *Pterospora andromedea*, another mycoheterotrophic member of the Ericaceae, where taxa with nitrogen-fixation potential constitute a substantial fraction of the endophytic bacterial community [41].

Predictive functional profiling further indicated high relative abundance of bacterial pathways associated with carbon fixation via the 3-hydroxypropionate cycle and glyoxylate assimilation in both compartments. These pathways are characteristic of chemoautotrophic or mixotrophic bacteria [42]. Considering that *M. uniflora* is achlorophyllous and relies on symbiotic associations for carbon acquisition, the presence of bacteria with predicted carbon-fixation potential raises the possibility that bacterial metabolism may contribute to local carbon transformations within the system, although direct metabolic exchange remains untested.

Based on our observations, a working hypothesis is proposed in which microbial metabolic pathways predicted from taxonomic inference could potentially generate glyoxylate intermediates within the system. The presence of calcium oxalate crystals in the *M. uniflora* root tissues [43], along with the high calcium concentrations (>7% dry weight; Table S2), is consistent with the possibility that oxalate-related metabolism contributes to host mineral homeostasis. Members of the Oxalobacteraceae family, representing ~5.25% of total bacterial abundance across compartments, have been reported to participate in oxalate turnover and could potentially influence carbon–mineral interactions within the holobiont [44]. In parallel, transcriptomic data from *M. hypopitys* [45] and unpublished data from *M. uniflora* indicate active expression of multiple glyoxylate-processing enzymes, suggesting that *Monotropa* species possesses the metabolic capacity to incorporate glyoxylate into multiple biochemical routes, including glycolate, glycerate, glycine and serine metabolism. In addition, fungal symbionts are known to secrete oxalic acid [46], providing an additional potential source of oxalate within the system. These observations support the hypothesis that glyoxylate could represent a metabolic intermediate linking microbial activity and host metabolism under carbon-limited conditions, although this possibility remains to be experimentally validated.

Although our data are consistent with this integrative hypothesis, several limitations must be considered. The number of biological replicates was limited, restricting statistical power and the ability to capture fine-scale spatial heterogeneity. Consequently, the ecological patterns described in this study should be interpreted as exploratory trends rather than definitive differences between compartments. The absence of bulk soil or surrounding tree rhizosphere samples limits the ability to determine whether the microbial communities detected in ECT are specifically associated with *M. uniflora* or reflect the broader forest soil microbiome and the rhizospheres of neighboring trees connected through ectomycorrhizal networks. Moreover, functional predictions derived from PICRUSt2, FAPROTAX, and FUNGuild are inherently inferential and do not reflect gene expression or enzymatic activity. Consequently, inferred pathways, particularly those uncommonly reported in rhizosphere ecosystems, should be interpreted cautiously. Experimental validation using multi-omics approaches and stable-isotope tracing will be essential to test the proposed metabolic interactions. In addition, a more detailed characterization of soil physicochemical properties together with nutrient analyses of multiple *M. uniflora* individuals would help clarify how edaphic variability influences plant nutrient composition and associated microbial communities. Soil chemistry strongly influences plant nutrient uptake, plant stoichiometry, and the structure of plant-associated microbiomes [47,48]. Such analyses could reveal whether variation in soil nutrient availability contributes to shaping plant stoichiometry and microbiome assembly in mycoheterotrophic systems.

Our results suggest that *M. uniflora* harbors structured microbial communities that tend to differ between external and internal compartments. These patterns likely reflect distinct microhabitats within the forest floor that influence microbial interactions and nutrient dynamics. Stable isotope evidence indicates that *M. uniflora* primarily acquires nitrogen through its fungal partners rather than through saprotrophic assimilation [49]. In addition, the presence of diazotrophic bacteria suggests that microbial communities associated with the plant may also contribute to nitrogen availability and nutrient cycling in the surrounding microenvironment [50,51]. Together, these findings expand current perspectives on mycoheterotrophic plants, positioning *M. uniflora* as an integrated component of belowground microbial ecosystems in which fungal and bacterial partners jointly influence carbon and nutrient dynamics.

## 4. Materials and Methods

### 4.1. Site Description and Sampling

Samples of *Monotropa uniflora* were collected in August 2023 from La Malinche National Park, Tlaxcala, Mexico (19°16′33.6″ N 98°02′28.4″ W ± 10 m). The park is located on the slopes of La Malinche Volcano, within the Transversal Volcanic Belt biogeographic province, in the Mexican transition zone between the Nearctic and Neotropical regions [52]. For microbiome analyses, two compartments were defined: (i) ECT, corresponding to soil surrounding *M. uniflora* and their associated mycorrhizal networks, and (ii) END, corresponding to tissues of the lower stem.

Biological replication was achieved using composite samples to reduce microscale spatial heterogeneity and capture a representative microbial signal from each compartment [53]. For the END compartment, three biological replicates were generated, each consisting of pooled lower stem tissue from three specimens (nine plants in total). Similarly, the ECT compartment comprised three replicates, each consisting of pooled soil collected from the immediate vicinity of plants within the same population patch. Replicates were obtained from plants separated by several meters.

Soil samples were collected using a stainless-steel spatula disinfected with 70% ethanol between samplings and transferred to sterile containers. Surface sterilization of plant tissue was performed as previously described [54]. Briefly, approximately 2 cm of the basal stem of each plant were excised, washed with sterile distilled water, immersed in 70% ethanol for 3 min, followed by 1% chlorine solution for 2 min, and rinsed three times with sterile distilled water. All samples were stored at −80 °C until DNA extraction.

### 4.2. Physicochemical Soil Analysis and Elemental Analysis of M. uniflora

Soil physicochemical properties and elemental composition of *M. uniflora* tissue were determined at the Central University Laboratory, Soil Department, Autonomous University of Chapingo (Texcoco, Mexico). A single composite sample was generated in each case, by pooling plant material or its associated ectomycorrhizospheric soil from nine individuals to capture the overall variability.

Soil pH was determined potentiometrically using a 1:2 soil-to-water ratio and electrical conductivity was measured using a conductivity bridge in the same suspension. Organic matter content was quantified using the Walkley–Black method [55], while inorganic nitrogen was extracted with 2N potassium chloride and quantified by steam distillation [56]. Available phosphorus was measured using the Bray P-1 method [57], whereas potassium was extracted with 1.0 N ammonium acetate (pH 7.0) at a 1:20 ratio and determined using flame emission spectrophotometry [58]. Calcium and magnesium were extracted with 1.0 N ammonium acetate and quantified using atomic absorption spectrophotometry [59]. Micronutrients (iron, copper, zinc, and manganese) were extracted using diethylenetriamine pentaacetate in a 1:4 ratio and analyzed by atomic absorption spectrophotometry [59], whereas boron was extracted with 1.0 M CaCl_2_ and colorimetrically quantified with azomethine-H [60]. Bulk density was determined using the core method [61], and soil texture was assessed using the Bouyoucos hydrometer method [62].

For plant tissue analysis, the samples were digested with a diacid mixture [63]. Nitrogen was quantified using the Kjeldahl method [64], whereas phosphorus was colorimetrically determined by molybdo-vanadate reduction [65]. Potassium was measured using flame emission spectrophotometry, while calcium, magnesium, iron, copper, zinc, and manganese were quantified using atomic absorption spectrophotometry [58]. Boron content was colorimetrically analyzed using azomethine-H [66].

### 4.3. Genomic DNA Extraction

Total genomic DNA was extracted from 100 mg of END samples using the BIOPURE Plant DNA Extraction Kit (BIOPURE, Mexico City, Mexico), following the manufacturer instructions. Prior to DNA extraction, samples were mechanically homogenized using a TissueLyser LT (Qiagen, Hilden, Germany) at 50 Hz for 2 min to facilitate the sample disruption. The homogenized material was then lysed using Solution A (Avr), a guanidine-based buffer, and incubated at 65 °C for 10–15 min according to the kit protocol. DNA was subsequently purified using silica spin columns and eluted following the manufacturer’s recommendations. For ECT samples, genomic DNA was extracted using the DNeasy PowerSoil Kit (Qiagen, Hilden, Germany) according to the manufacturer’s instructions. DNA integrity was evaluated using 0.8% agarose gel electrophoresis, and DNA concentration was measured using a NanoDrop One spectrophotometer (Thermo Fisher Scientific, Waltham, MA, USA).

### 4.4. Library Construction and Bioinformatic Analysis

Bioinformatic analyses were performed as previously described [67], with minor modifications. Bacterial communities were characterized by amplifying the V3–V4 hypervariable region of the 16S rRNA gene using the primer pair 357F and 783R [68,69]. Fungal communities were targeted using primers ITS1F [70] and ITS2 [71]. Amplicon libraries were generated using the Illumina 16S Metagenomic Sequencing Library Preparation protocol (Illumina, San Diego, CA, USA), using Herculase II Fusion DNA Polymerase (Agilent, Santa Clara, CA, USA) and Nextera XT indices (Illumina, San Diego, CA, USA) and sequenced on the Illumina MiSeq platform (2 × 300 bp) (Illumina, San Diego, CA, USA) with a 30% PhiX spike-in (Illumina, San Diego, CA, USA). Library preparation and sequencing were performed by Macrogen Inc. (Seoul, Republic of Korea).

Microbiome bioinformatic analyses were conducted using qiime2-amplicon-2024.5 [72]. Amplicon sequence variants (ASVs) were inferred using the DADA2 denoising algorithm implemented in QIIME2. Taxonomic assignments were performed using the SILVA v138.1 database for bacterial 16S rRNA sequences and the UNITE v9 database for fungal ITS sequences. Additionally, ASVs were manually curated based on BLAST v2.17.0 searches against the NCBI rRNA/ITS reference databases to refine taxonomic assignments.

For fungal communities, trophic modes and ecological guilds were inferred using FUNGuild v1.1 [73]. Bacterial functional potentials were inferred using FAPROTAX v1.2.1 [74] and PICRUSt2 v2.4.2 [75]. FUNGuild and FAPROTAX were implemented through the microeco v2.0.0 R package using the trans_func workflow [76]. These approaches infer predicted functional potential based on taxonomic profiles and phylogenetic inference and do not represent direct measurements of metabolic activity or gene expression.

### 4.5. Statistical Analysis

All statistical analyses and visualizations were conducted in R v4.4.2 using the qiime2R v0.99.6, phyloseq v1.48.0, vegan v2.7-3, tidyverse v2.0.0, ggforce v0.5.0, factoextra v2.0.0, DESeq2 v1.44.0, ape v5.8-1, microbiome v1.26.0, heatmaply v1.6.0, ggsignif v0.6.4, plotly v4.12.0, pheatmap v1.0.13, ggmap v4.0.1, sf v1.0-21, rnaturalearth v1.0.1, rnaturaldata v1.0.0, ggspatial v1.1.9, and ggrepel v0.9.6 packages. Analyses were performed separately for bacterial (16S rRNA) and fungal (ITS) datasets. Alpha diversity was assessed using richness-based (Observed richness, Chao1, ACE, and Fisher) and diversity indices incorporating evenness (Shannon and Simpson). Differences between compartments (ECT and END) were evaluated using the Wilcoxon rank-sum test. Beta diversity was evaluated using Bray–Curtis dissimilarities and ordination was performed by Principal Coordinates Analysis (PCoA). Statistical differences in community composition between compartments were determined using PERMANOVA (adonis2 function) with 999 permutations. The reported R^2^ values correspond to unadjusted explained variance. Homogeneity of multivariate dispersion was assessed using the betadisper function in the vegan package followed by permutation tests to evaluate whether differences in community structure could be influenced by unequal within-group dispersion. ANOSIM was applied as an additional, exploratory measure of compositional separation between groups.

Differential abundance analyses were performed using DESeq2, applied to unrarefied count data following variance stabilization. Taxa were considered differentially abundant when the adjusted *p*-value (FDR) was <0.05. Analyses were conducted independently at multiple taxonomic ranks (phylum to genus) for bacterial and fungal communities. Heatmaps of significantly differentially abundant genera were generated based on variance-stabilized abundances and scaled by row to highlight relative abundance patterns across samples. Hierarchical clustering was applied to both taxa and samples. Statistical comparisons of predicted functional categories between compartments were conducted using non-parametric tests (Wilcoxon rank-sum test). The geographic outline of Mexico was obtained from the Natural Earth database: https://www.naturalearthdata.com.

## 5. Conclusions

This study suggested compartmentalization of bacterial and fungal communities associated with the ECT and END tissues of *M. uniflora*, indicating that microbial community structure in this system extends beyond the primary mycorrhizal partners traditionally associated with mycoheterotrophy. The ECT was enriched with saprotrophic fungi and metabolically diverse bacteria linked to nutrient transformations, whereas the END compartment harbored bacterial taxa potentially adapted to internal, low-oxygen plant environments.

Predictive functional analyses suggested that these microbial communities possess metabolic potential related to nitrogen transformations and carbon-associated pathways, positioning bacteria as possible contributors to nutrient dynamics in the *M. uniflora* microenvironment. However, these functional predictions are based on amplicon-derived inference and require experimental validation; thus, they should be interpreted as initial characterization. Despite certain limitations such as a limited number of biological replicates and the lack of additional control compartments like bulk soil or autotrophic plant endospheres, these findings advance the understanding of microbiome structures in mycoheterotrophic systems. This study reframes mycoheterotrophic plants as integrated components of complex belowground microbial networks, rather than passive recipients of fungal-derived carbon. This approach provides a framework for future studies aimed at experimentally testing microbial contributions to nutrient cycling and plant nutrition in forest ecosystems.

## Data Availability

Research data supporting this publication is available from the Sequence Read Archive (SRA) of the National Center for Biotechnology Information (NCBI) under BioProject accession PRJNA1375162.

## Supplementary Materials

The following supporting information can be downloaded at: https://www.mdpi.com/article/10.3390/plants15081145/s1, Figure S1: Rarefaction curves showing ASV richness of (a) fungal and (b) bacterial communities associated with *M. uniflora* in two compartments: lower stem endosphere (END) and ectomycorrhizospheric soil (ECT); Figure S2: Beta-dispersion analysis based on Bray–Curtis distances for bacterial (a) and fungal (b) communities associated with the ectomycorrhizospheric soil (ECT) and lower stem endosphere (END) of *M. uniflora*. Figure S3: Heatmap of differentially abundant genera between the ectomycorrhizospheric soil (ECT) and lower stem endosphere (END) of *M. uniflora*. Table S1: Summary of read filtering steps for 16S and ITS gene markers across samples from *M. uniflora*; Table S2: Physicochemical analysis of the ectomycorrhizospheric soil of *M. uniflora*; Table S3. Macro- and micronutrient composition of *Monotropa uniflora* plant tissue; File S1.

## Abbreviations

The following abbreviations are used in this manuscript:

16S rRNA	16S ribosomal RNA
ANOSIM	Analysis of Similarities
ASVs	Amplicon sequence variants
END	Lower stem endosphere
FAPROTAX	Functional Annotation of Prokaryotic Taxa
FDR	False Discovery Rate
ITS	Internal transcribed spacer
PCoA	Principal Coordinates Analysis
PICRUSt2	Phylogenetic Investigation of Communities by Reconstruction of Unobserved States
PERMANOVA	Permutational Multivariate Analysis of Variance
