# Supplementary material for: Bacterial and Fungal Communities Associated with the Ectomycorrhizospheric Soil and Stem Endosphere of the Mycoheterotrophic Plant Monotropa uniflora
Source: Plants (Basel). 2026 Apr 8;15(8):1145. doi: 10.3390/plants15081145 (PMC13119174; doi:10.3390/plants15081145)
Supplement: Supplementary file 1 [file plants-15-01145-s001.zip › Supplementary Material_v2.pdf]

Bacterial and fungal communities associated with the ectomy-corrhizospheric soil and stem endosphere of the mycohetero-trophic plant *Monotropa uniflora*

Leandro Alberto Núñez-Muñoz [1], Brenda Yazmín Vargas-Hernández [1], Melissa Cheryn García-Sierra [1], Berenice Calderón-Pérez [1], Beatriz Xoconostle-Cázares [1,2,*], and Roberto Ruiz-Medrano [1,2,*]

[1] Departamento de Biotecnología y Bioingeniería, Centro de Investigación y de Estudios Avanzados. Av. Instituto Politécnico Nacional 2508, Col. San Pedro Zacatenco, 07360 CDMX, Mexico; leandro.nunez@cinvestav.mx (LAN-M); byvargas@cinvestav.mx (BYV-H); melissa.garcia@cinvestav.mx (MCG-S); bcalderon@cinvestav.mx (BC-P).

[2] Programa de Doctorado Transdiciplinario en Desarrollo Científico y Tecnológico para la Sociedad, Centro de Investigación y de Estudios Avanzados. Av. Instituto Politécnico Nacional 2508, Col. San Pedro Zacatenco, 07360 CDMX, Mexico.

* Correspondence: bxoconos@cinvestav.mx (BX-C); rmedrano@cinvestav.mx (RR-M); Tel.: (+52) 5557473800 Ext. 4315 and 4301.

Supplementary Material

Supplementary Figure

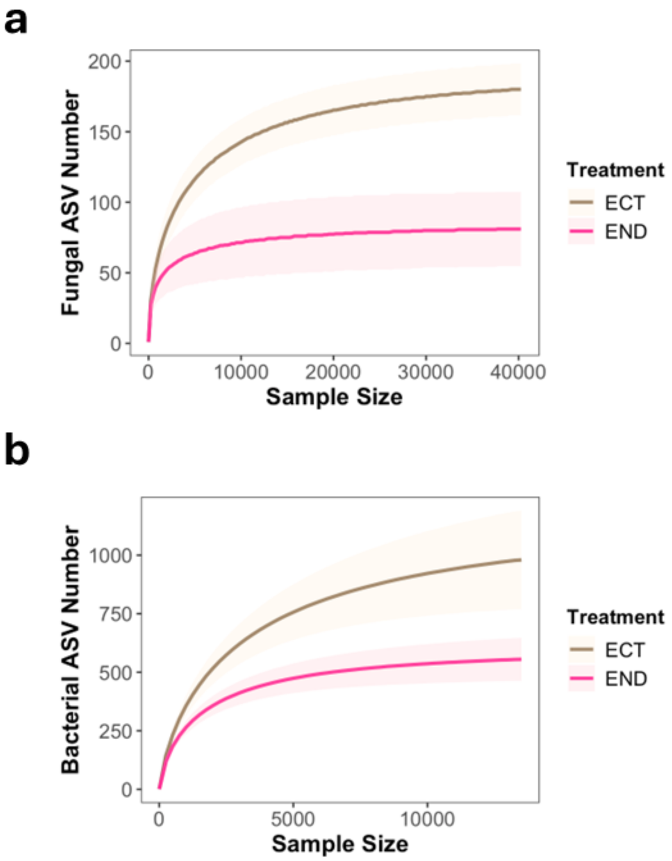

Figure S1. Rarefaction curves showing ASV richness of (**a**) fungal and (**b**) bacterial communities associated with *M. uniflora* in two compartments: lower stem endosphere (END) and ectomycorrhizospheric soil (ECT).

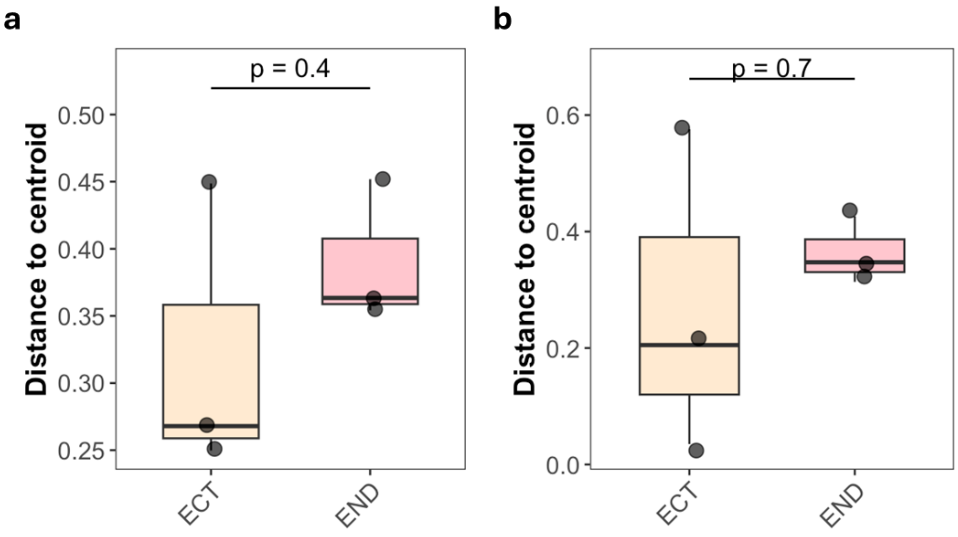

Figure S2. Beta-dispersion analysis based on Bray–Curtis distances for bacterial (**a**) and fungal (**b**) communities associated with the ectomycorrhizospheric soil (ECT) and lower stem endosphere (END) of *M. uniflora*.

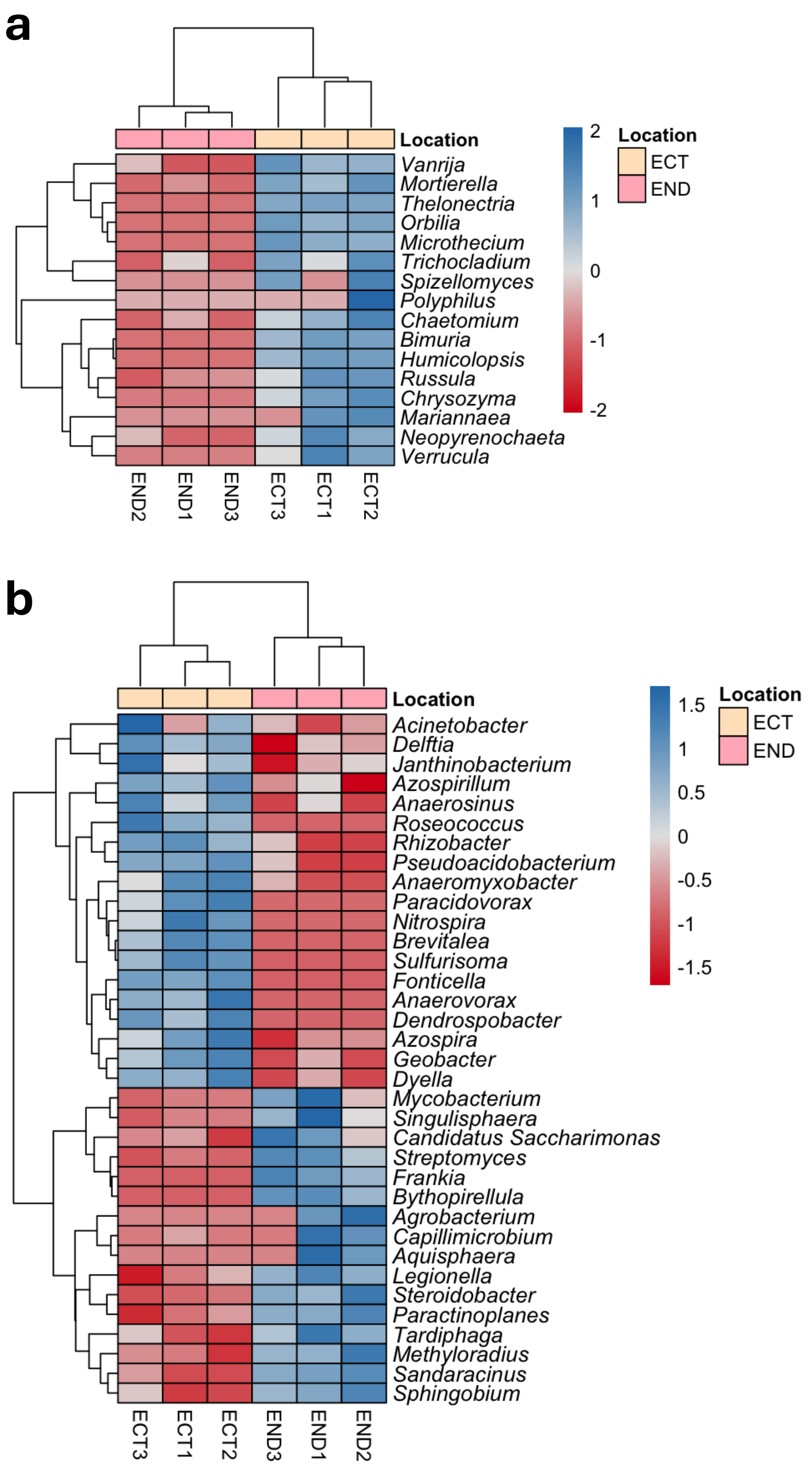

Figure S3. Heatmap of differentially abundant genera between the ectomycorrhizospheric soil (ECT) and lower stem endosphere (END) of *M. uniflora*. Variance-stabilized abundances of fungal (**a**) and bacterial (**b**) genera identified as significantly enriched between ECT and END compartments by DESeq2 analysis (adjusted $p < 0.05$). Values were scaled by row (z-score) to highlight relative abundance patterns across samples.

Supplementary Tables

Table S1. Summary of read filtering steps for 16S rRNA and ITS gene markers across samples from *M. uniflora.*

Gene Marker	Sample	Input	Filtered	Denoised	Merged	Non-chimeric
16S	**END-1**	84,765	67,480	66,752	65,204	63,803
	**END-2**	96,660	75,539	75,037	73,833	72,748
	**END-3**	90,286	72,380	71,568	70,360	68,888
	**ECT-1**	65,603	48,520	42,531	32,277	30,588
	**ECT-2**	103,506	86,624	83,530	77,315	73,370
	**ECT-3**	86,458	71,483	66,411	51,504	43,608
	**Mean**	87,879.7	70,337.7	67,638.2	61,748.8	58,834.2
	**Mean (%)**	100.0	80.0	77.0	70.3	66.9
ITS	**END-1**	95,195	80,055	79,952	79,016	79,011
	**END-2**	99,656	80,542	80,291	79,403	78,737
	**END-3**	118,313	97,692	97,564	97,354	97,278
	**ECT-1**	97,202	80,468	80,291	79,268	79,186
	**ECT-2**	76,054	65,778	65,550	64,680	64,613
	**ECT-3**	117,889	99,268	99,085	97,846	97,715
	**Mean**	100,718.2	83,967.2	83,788.8	82,927.8	82,756.7
	**Mean (%)**	100.0	83.4	83.2	82.3	82.2

Table S2. Physicochemical analysis of the ectomycorrhizospheric soil of *M. uniflora.*

Parameter	Value
pH	6.62
Electrical conductivity (dS/m)	0.10
Organic matter (%)	7.10
Inorganic nitrogen (mg/kg)	26.3
Phosphorus (mg/kg)	243.96
Potassium (mg/kg)	2064
Calcium (mg/kg)	5865
Magnesium (mg/kg)	864
Iron (mg/kg)	92.63
Copper (mg/kg)	0.94
Zinc (mg/kg)	8.91
Manganese (mg/kg)	56.87
Boron (mg/kg)	2.21
Bulk density (g/cm^3)	0.53
Sand (%)	34.2
Silt (%)	43.3
Clay (%)	22.5
Texture	Loam

Table S3. Macro- and micronutrient composition of *Monotropa uniflora* plant tissue.

Nutrient Type	Parameter	Value
Macronutrients (%)	Nitrogen	2.01
	Phosphorus	0.24
	Potassium	3.85
	Calcium	7.63
	Magnesium	1.73
Micronutrients (mg/kg)	Iron	1449.75
	Copper	18.25
	Zinc	77.50
	Manganese	52.50
	Boron	26.24
